# Improving social gaze behavior in fragile X syndrome using a behavioral skills training approach: a proof of concept study

**DOI:** 10.1186/s11689-018-9243-z

**Published:** 2018-08-28

**Authors:** Caitlin E. Gannon, Tobias C. Britton, Ellen H. Wilkinson, Scott S. Hall

**Affiliations:** 1 Pacific Graduate School of Psychology, Palo Alto University, Palo Alto, California USA; 2 Department of Psychiatry and Behavioral Sciences, Stanford University School of Medicine, Stanford, California USA

**Keywords:** Fragile X syndrome, Social gaze behavior, Behavioral treatment

## Abstract

**Background:**

Individuals diagnosed with fragile X syndrome (FXS), the most common known inherited form of intellectual disability, commonly exhibit significant impairments in social gaze behavior during interactions with others. Although this behavior can restrict social development and limit educational opportunities, behavioral interventions designed to improve social gaze behavior have not been developed for this population. In this proof of concept (PoC) study, we examined whether administering a behavioral skills training package—discrete trial instruction (DTI) plus relaxation training—could increase social gaze duration in males with FXS.

**Methods:**

As part of a larger clinical trial, 20 boys with FXS, aged 8 to 18 years, were randomized to receive DTI plus relaxation training administered at one of two prescribed doses over a 2-day period at our research center. Potential improvements in social gaze behavior were evaluated by direct observations conducted across trials during the training, and generalization effects were examined by administering a social challenge before and after the treatment. During the social challenge, social gaze behavior was recorded using an eye tracker and physiological arousal levels were simultaneously recorded by monitoring the child’s heart rate.

**Results:**

Levels of social gaze behavior increased significantly across blocks of training trials for six (60%) boys who received the high-dose behavioral treatment and for three (30%) boys who received the low-dose behavioral treatment. Boys who received the high-dose treatment also showed greater improvements in social gaze behavior during the social challenge compared to boys who received the low-dose treatment. There was no effect of the treatment on physiological arousal levels recorded on the heart rate monitor at either dose.

**Conclusions:**

These results suggest that appropriate social gaze behavior can be successfully taught to boys with FXS using a standardized behavioral skills training approach. Future studies will need to evaluate whether younger children with FXS might benefit from this treatment, and/or whether more naturalistic forms of behavioral skills training might be beneficial, before social gaze avoidance becomes established in the child’s repertoire.

**Trial registration:**

ClinicalTrials.gov, NCT02616796. Registered 30 November 2015.

## Background

The ability to engage in appropriate social gaze behavior with others is arguably one of the most important skills in the development of human social and communication functioning. Extensive work has determined that maintaining appropriate social gaze is a critical prerequisite for language development, emotion recognition, social engagement, and general learning through joint attention [1–4]. Maintaining social gaze with others not only serves to regulate face-to-face social interactions during early social development, but also facilitates the later coordination of visual attention between individuals or objects of interest [4, 5]. The development of appropriate social gaze behavior can therefore be considered a critical target for behavioral interventions for children with developmental disabilities.

A subgroup of children with developmental disabilities who are particularly prone to exhibit impairments in social gaze behavior are individuals with fragile X syndrome (FXS). FXS is caused by mutations to the *FMR1* gene at locus 27.3 on the long arm of the X chromosome [6] which results in excessive methylation of the gene and subsequent reduced or absent fragile X mental retardation protein (FMRP). Characteristic phenotypic features of the disorder include impairments in intellectual functioning [7] and an increased risk for autistic-like behaviors (e.g., social avoidance, communication impairments, and repetitive behaviors) [8, 9]. A hallmark behavioral feature of children with FXS is a propensity to avoid social gaze during demanding social situations [10]. Studies have shown that boys with FXS typically engage in increased social gaze avoidance and social withdrawal during social interactions with unfamiliar people compared to individuals who are more familiar [10–13]. In a naturalistic eye-tracking study, for example, Hall and colleagues [14] reported that individuals with FXS spent less time looking at an unfamiliar experimenter and exhibited shorter inter-episodes of social gaze when compared to age and IQ-matched controls [14].

In addition to the characteristic behavioral phenotype of eye gaze avoidance in individuals diagnosed with FXS, data from neuroimaging studies have shown that these individuals exhibit neural circuitry abnormalities in brain regions associated with social behavior. For example, Bruno and colleagues showed that when individuals with FXS were required to look at static photographs of faces in the scanner, neural signaling across multiple brain regions was slower to habituate compared to age and IQ-matched controls [15]. Another study conducted by Garrett and colleagues demonstrated that individuals with FXS exhibited decreased activation in brain regions known to respond to face and gaze stimuli (i.e., fusiform gyrus and superior temporal sulcus) [16].

Despite notable behavioral and neural circuitry deficits in social functioning exhibited by individuals with FXS, there is a long-standing debate among practitioners as to whether interventions should be implemented to improve social gaze behavior in FXS. For example, Scharfenaker, O’Conner, Stackhouse, Braden, and Gray [17] issued the following guidelines to practitioners who implement interventions for individuals with FXS: “Allow avoidant eye gaze. Don’t insist on eye contact” (p.371) and “Position the child so eye contact is not always demanded” (p. 387). Similarly, in their guide for behavioral practitioners working with children with FXS, Morris and colleagues contended that requiring eye contact may result in a number of drawbacks, including “signs of anxiety, more gaze aversion, and possible escalation of problematic behavior” [18] (p.99). These guidelines may, in part, have resulted from observations that individuals with FXS, particularly males, exhibit elevated levels of physiological arousal (termed “hyperarousal”) when required to complete demanding tasks or to engage in social interactions with others [8, 9, 12, 19]. Although this association has often been reported in the literature, recent research indicates that hyperarousal in FXS may not necessarily be associated with social interaction. In a recent review of studies conducted on individuals with FXS, for example, Klusek and colleagues [20] have suggested that the potential increases in physiological arousal that occur in response to social or cognitive stressors in FXS may, in fact, be of similar magnitude to that seen in controls. Rather, these authors suggest that hyperarousal in FXS may be the result of a chronic underlying physiological state rather than a context-dependent phenomenon. Individuals with FXS may therefore be responding to social or cognitive stressors at levels appropriate to the situation [9, 21, 22].

If this is the case, failing to teach appropriate social gaze behavior to individuals with FXS could ultimately be detrimental to the person’s social development, as well as significantly reducing access to future educational opportunities. For example, several studies have indicated that engaging in frequent social gaze avoidance can negatively impact social interaction skills and communication flow, given that crucial non-verbal gestures and facial expressions that usually aid social interaction will be missed [23, 24]. Devising interventions that could target appropriate social gaze behavior in FXS, without increasing social gaze avoidance and anxiety, would therefore appear to be particularly important.

One such method that could facilitate the acquisition of social skills in individuals with FXS is discrete trial instruction (DTI)—a standardized teaching procedure that utilizes individualized instruction, well-defined steps, and a consistent rate of training trials to enhance learning [25, 26]. Indeed, DTI can be advantageous precisely because it involves administering multiple learning opportunities at a relatively high rate—roughly 50–100 per hour [27]—therefore maximizing the learner’s exposure to the relevant contingencies and ultimately facilitating acquisition of the targeted skill over a relatively short period of time.

DTI has been successfully employed to teach a variety of skills to children diagnosed with autism spectrum disorder (ASD), including imitation [28], play, vocal, and non-vocal communication, as well as social skills [29]. Several previous studies have also demonstrated that DTI can be effective for teaching appropriate social gaze behavior to children with ASD or intellectual disabilities [29–31]. In an early study conducted by Foxx [29], for example, three children with ASD or intellectual disabilities, aged 6 to 8 years, were taught to maintain eye contact with a therapist using DTI. On each trial, the therapist held up an edible item to the eye region and delivered a verbal prompt “Look at me.” If a participant engaged in eye contact, the therapist then delivered the edible item and verbal praise. If the participant failed to make eye contact within 5 s, the therapist implemented an overcorrection procedure by verbally and physically prompting the participant to move his head in one of three directions for a fixed period of time. For all three children, social gaze duration with the therapist improved.

In a study by Cook and colleagues [30], 21 children with ASD, aged 3 to 12 years old, were each exposed to a progressive training model designed to increase instructional control of social gaze. The model included the following phases: contingent praise only, contingent edibles plus praise, stimulus prompts plus contingent edibles and praise, and contingent video and praise. Results from this study demonstrated that using this procedure allowed the therapists to thin the reinforcement schedule (e.g., from edibles to praise) to some extent in all participants and to maintain learning at or above 80% for 17 of the participants.

Finally, Rapp and colleagues [31] further modified the protocol described by Cook et al. [30] to improve social gaze behavior in 15 children with ASD, aged 3 to 7 years. If social gaze was not acquired using the standard progressive model, one or more of the following modifications were made: prompts to sit, altered or skipped phases, altered prompts, or mastery criteria at 70% or higher for five consecutive sessions. Results indicated that social gaze improved for 11 of the 15 participants using the progressive model, but that for 8 participants, one or more procedural changes were required for the children to acquire eye contact. Four participants were unable to acquire social gaze despite modifications to the treatment protocol.

In both the Cook et al. [30] and Rapp et al. [31] studies, however, the dependent variable was the latency to engage in social gaze rather than the duration of social gaze. Thus, in both of those studies, it is unclear to what extent the children actually exhibited social gaze behavior, once instructional control had been achieved. To date, only one study has been conducted to examine whether social gaze duration can be improved in children with FXS. Hall, Maynes, and Reiss [32] employed DTI to teach six boys with FXS, aged 8 to 17 years to engage in increasingly longer durations of social gaze during social interactions with a therapist over a 2-day period. In the Hall et al. study, boys with FXS were asked to look while the therapist held an edible item close to his eye in a series of discrete trials, similar to the procedure utilized by Foxx [29]. As the trials progressed, a percentile schedule of reinforcement stipulated whether longer durations of social gaze would meet the criteria for reinforcement. For three participants, an overcorrection procedure similar to the procedure employed by Foxx [29] was required, given that those children did not initially exhibit appropriate social gaze when prompted at the beginning of training. Results from this study demonstrated that although each participant varied in improvement, for 5 of the 6 participants, social gaze duration increased steadily across trials. This suggested that contrary to guidelines issued by some practitioners, behavioral skills training resulted in improved eye contact duration in FXS, at least in the short term.

### The present study

In the present study, we refined the teaching paradigm utilized by Hall and colleagues [32] in a number of ways to facilitate the teaching of appropriate social gaze behavior to boys with FXS. First, to address the potential for elevated autonomic nervous system reactivity (i.e., “hyperarousal”) commonly reported in males with FXS, we paired the DTI procedure with stress- and anxiety-reduction relaxation training. In addition, we used verbal and physical prompts rather than an overcorrection procedure to facilitate the initiation of social gaze and employed contingent verbal praise and a token economy system rather than immediate delivery of an edible item to reinforce appropriate social gaze. To examine the dosage effect of the treatment, we randomized participants to receive the treatment at one of two dose levels: *high* or *low*. Finally, to examine whether improvements in social gaze duration might generalize to a naturalistic social encounter with an unfamiliar person, participants received a brief social challenge before and after treatment.

We had three research questions:

1. To what extent would administration of DTI plus relaxation training result in improved social gaze duration in boys in FXS?
2. To what extent would exposure to a higher dose of treatment result in greater improvements in social gaze duration in boys in FXS?
3. To what extent would exposure to the treatment result in improved social gaze behavior and decreased physiological arousal during a subsequent naturalistic social encounter?

## Method

### Participants and setting

Participants were recruited via an online screening survey sent out to members of the National Fragile X Foundation. The cover page of the survey stated: “We are conducting a study to evaluate a brief social skills intervention for boys with fragile X syndrome (FXS), ages 8-18 years. Eligible participants will be required to travel to Stanford University for a 4-day visit. To determine your son’s eligibility for the study, please complete the brief survey by clicking on the arrow at the bottom of the page”. The survey obtained basic demographic information concerning the child’s age, diagnosis, whether the child had a neurological and/or sensory impairment, whether the child was taking any medications, and whether the child exhibited any problem behaviors (e.g., aggression, self-injurious behavior, elopement, property destruction, or stereotyped behavior). Caregivers also completed the Eye Contact Avoidance Scale (ECAS), a 15-item questionnaire designed to quantify social gaze avoidance in individuals with developmental disabilities [11].

Boys with FXS were included in the present study if they were aged between 8 and 18 years old, had a confirmed diagnosis of FXS (> 200 CGG repeats on the *FMR1* gene), and had obtained a total score greater than 30 points on the ECAS [11]. Participants were excluded if caregivers indicated that their son had other neurological or sensory impairments (e.g., head trauma and blindness), if they engaged in frequent and/or severe problem behaviors, or if they had any other known medical, psychiatric, or behavioral conditions that would preclude participation in the study. Twenty-one boys with FXS met the study inclusion criteria, but one boy was unable to participate in the study because he engaged in frequent elopement. Twenty boys with FXS were therefore included in the present study.

Demographic information for each participant is provided in Table 1.

**Table 1:** Demographic characteristics of the sample

Subject number	Age (years)	IQ^a^	Medications	ADOS module^b^	ADOS classification^b^	ADOS comparison score^b^	ECAS total score^c^
P1	15.3	57	No	Fluent speech	Autism	7	42
P2	12.1	68	No	Fluent speech	Autism	8	48
P5	15.2	54	Yes	Fluent speech	Non-spectrum	1	44
P6	13.8	56	Yes	Fluent speech	ASD	4	33
P9	16.2	42	Yes	Phrase speech	Non-spectrum	3	46
P12	15.9	46	Yes	Single words	ASD	4	50
P13	11.6	56	Yes	Fluent speech	Autism	7	43
P14	8.9	62	No	Fluent speech	Autism	7	41
P15	10.8	65	No	Fluent speech	Autism	8	54
P17	9.9	59	Yes	Fluent speech	Autism	9	40
P18	18.5	41	No	Phrase speech	Non-spectrum	3	45
P21	13.8	43	No	Fluent speech	Autism	7	43
P22	14.8	40	No	Phrase speech	Autism	9	38
P25	15.5	84	Yes	Fluent speech	Autism	7	47
P27	10.6	69	Yes	Fluent speech	Autism	10	47
P30	15.2	40	Yes	Phrase speech	Autism	9	41
P33	9.9	63	Yes	Phrase speech	Autism	8	41
P34	10.6	50	Yes	Phrase speech	Autism	8	39
P37	9.8	57	No	Phrase speech	Autism	7	40
P40	9.2	40	Yes	Phrase speech	Autism	7	37

^a^Weschler Abbreviated Scale of Intelligence, Second Edition (WASI-II)

^b^Autism Diagnostic Observation Schedule, Second Edition (ADOS-2)

^c^Eye Contact Avoidance Scale (ECAS)

The mean age of the participants was 12.9 years (*SD* = 2.9 years, range = 8 to 18 years), and the mean full-scale IQ was 54.6 (*SD* = 12.0, range = 40 to 84). Twelve (60%) boys were taking psychoactive medications including stimulants, antidepressant medications, and antipsychotic medications. On the Autism Diagnostic Observation Schedule, 2nd Edition (ADOS-2) [33], 15 (75%) boys were classified in the “autism” range, 2 (10%) boys were classified in the “ASD” range, and 3 (15%) boys were classified in the “non-spectrum” range. Module 1 (single words) was administered to 1 (5%) boy, module 2 (phrase speech) was administered to 8 (40%) boys, and module 3 (fluent speech) was administered to 11 (55%) boys.

Sessions were conducted in a room that contained a table or desk, chairs, and highly-to-moderately preferred tangible items and activities as identified using the multiple stimulus without replacement (MSWO) protocol described by DeLeon and Iwata [34]. All experimenters and observers who participated in the study had previous experience in the use of behavioral interventions with developmentally disabled children. In addition, specific training activities were employed to ensure that staff could reliably observe behavior and respond appropriately during sessions. Each staff member received written instructions describing the observation procedures and experimental protocol described below. After reading and reviewing these materials with an experienced staff member, a new staff member was assigned to conduct informal observations, reliability observations, and primary data observations for approximately five sessions each. Persons serving as experimenters and observers (i.e., those conducting sessions) did so only after demonstrating competence as an observer. At least one experienced staff member was present during each session and provided feedback regarding compliance with the procedures as needed.

### Procedure

Each participant visited our research center on four consecutive days. On day 1, a female examiner administered a social challenge to measure baseline levels of social gaze behavior and physiological arousal. On days 2 and 3, a trained behavior therapist administered the targeted behavioral treatment. Finally, on day 4, a different female examiner administered the social challenge to evaluate potential generalization effects in social gaze behavior and physiological arousal.

#### Social challenge

The social challenge was administered according to the procedures detailed in Hall and colleagues [14]. During the social challenge, the participant sat in a chair directly opposite to a female examiner and a Tobii X120 eye tracker, positioned on a table between the participant and the examiner, directly recorded the participant’s social gaze behavior. The Tobii X120 was chosen because it is a stand-alone system that allows a freedom-of-head movement of 30 × 22 × 30 cm and enables a custom setup to be employed. To enable a naturalistic converation during the social challenge, a scene camera was positioned directly above and behind the participant’s chair to monitor the face of the examiner and a mini user -camera was positioned centrally on top of the eye tracker to monitor the face of the participant. In order to calibrate the eye tracker to the area where the participant would be looking at the examiner’s face, a standard 5-point calibration procedure employed in Tobii Studio was displayed to the participant on a computer monitor that was subsequently removed before the experiment began. After successful calibration, the examiner then positioned her head so that it was centered in the same plane as to where the computer monitor had been positioned. To check for calibration accuracy, the examiner asked the subject to briefly look at her chin, nose, mouth, and each ear respectively prior to beginning the social challenge. A research assistant simultaneously monitored the accuracy of the eye tracking using the Live Viewer feature in Tobii Studio while the data were being collected. The research assistant also ensured that the participant’s head did not move out of the range of the eye tracker during the social challenge by using verbal reminders if necessary. All eye movement and video/audio data were collected on a Dell Precision laptop computer using Tobii Studio 3.0 software operated by the research assistant. During the social challenge, the examiner engaged the participant in a conversation for 5 min by asking the participant a series of questions pertaining to the child’s interests and reminding the participant to maintain eye contact every 30 s. The dependent variable was the percentage of time that the participant engaged in social gaze with the examiner. One participant (P17) did not complete the social challenge on day 1 or day 4 due to a technical error with the eye tracker during his visit. The data for this participant were therefore not included in the analyses of the eye-tracking data.

To provide a quantitative measure of physiological arousal, each participant wore a Polar chest belt or wrist-based heart rate monitor during the social challenge. The dependent variable was the participant’s heart rate, recorded in beats per minute (bpm). For two participants (P17 and P18), heart rate data were not collected due to technical errors and one participant (P33) was unable to tolerate wearing the heart rate monitor. Data for these participants were therefore not included in the analyses of the heart rate data.

#### Randomization

Following the baseline social challenge on day 1, participants were randomized to receive the targeted behavioral treatment at one of two “dose” levels: high dose or low dose. Participants randomized to the high-dose group were scheduled to receive a total of 320 DTI trials across days 2 and 3, conducted in eight 1-h blocks. Participants randomized to the low-dose group were scheduled to receive a total of 160 DTI trials across days 2 and 3, conducted in four 1-h blocks. For participants in the low-dose group, 1-h training blocks were alternated with 1-h blocks of unstructured play*.* Each session of unstructured play consisted of engaging in games that were social in nature; however, no formal academic or social demands were placed on the participant. Thus, although the total number of training trials differed between groups, participants in each group were exposed to similar amounts of therapist time (approximately 8 h) on days 2 and 3. Ten boys were randomized to the high-dose group and ten boys were randomized to the low-dose group. [Note that two boys who received high-dose treatment received only 240 training trials rather than the scheduled 320 trials. This was because these boys required longer breaks between sessions than the other participants].

#### Relaxation training

Prior to beginning each session of DTI, the therapist introduced a variety of relaxation exercises using deep breathing and progressive muscle relaxation techniques [35, 36]. These exercises were employed to reduce any potential physiological arousal issues during DTI. Exercises were introduced in the following manner:

Today we will be practicing how to calm our bodies. Sometimes when we talk to new people or have to do something that makes us feel scared, we might feel our hearts beating faster, our legs turning to jello, or even butterflies in our stomachs. By taking deep breaths and moving our bodies in different ways, our bodies are able to calm down and relax. I’m going to show you how to do it and then it will be your turn to try! Let’s get started!

The therapist employed a behavioral skill model of training by providing a verbal and pictorial description of six different exercises to the participant, modeling the exercises, prompting the participant to engage in the exercises, and providing feedback until the participant could complete each of the exercises.

Participants were shown a laminated card containing icons for a *pufferfish*, *snowman*, *turtle*, *cat*, *batman*, and *lemon,* on the one side, and step-by-step instructions for each exercise on the back. Participants could choose to perform from any three of the six exercises (or could repeat the same exercise three times). Following the relaxation exercises, the therapist reviewed a list of guidelines with the participant concerning how to appropriately listen and/or speak to someone.

#### Baseline DTI trials

Participants were required to sit in a chair directly facing the therapist and to engage with the therapist in a series of baseline discrete trials. On *look while listening* trials, the therapist stated, “I’m going to tell you about something…remember to look at my eyes while I talk to you” to signal the beginning of the condition. Topics included the therapist telling a story, “Let me tell you about the time I went to…Let me tell you about the movie I saw this weekend…”, or providing information, such as discussing the steps to making a peanut butter and jelly sandwich. If the participant began to talk in response to what the therapist was talking about, the participant was allowed to continue and was responded to as he/she would in a natural conversation. On *look while speaking* trials, the therapist stated, “I want to learn more about you/I want you to tell me about…remember to look at my eyes while you talk to me.” The therapist asked the participant questions about things they liked, activities they enjoyed doing, school (teacher, favorite subjects, friends, sports, etc.), their favorite foods, their family, where they live, etc. Depending on the participant’s verbal abilities, the therapist would ask open-ended questions to encourage more conversation. The therapist encouraged the participant to ask questions as well. While almost all participants could speak in full sentences at least some of the time, the therapist accommodated the needs of those who rarely spoke in full sentences by asking simple questions that required either one word or yes/no responses. On each trial, the therapist delivered a verbal prompt to engage the participant in social interaction (i.e., providing the child with information or asking the child questions as described above) but no reinforcement was provided for social gaze during baseline trials. Blocks of 10 *look while listening* trials were alternated with blocks of 10 *look while speaking* trials.

#### Training trials

During training trials, engaging in social gaze was reinforced with praise (e.g., “Good job!”), tokens, and access to leisure materials upon mastery of criteria set by a percentile schedule of reinforcement (see below). If the child failed to engage in social gaze, or duration of social gaze did not meet criteria for reinforcement, increased verbal and gestural prompting was used to promote social gaze on the next trial. Verbal prompting included reminders such as, “Remember to keep looking at me while I talk,” before a new verbal prompt was delivered. If no social gaze occurred within 5 s of the stimulus prompt, the therapist used a least-to-most prompting hierarchy that included gestural prompts, gestural/verbal prompts, and gestural/verbal/physical prompts. For gestural prompts, the therapist pointed both fingers in the direction of the participant’s eyes and moved both fingers to point at his/her eyes. For verbal prompts, the therapist provided a verbal reminder for the participant to engage in appropriate social gaze. For physical prompts, the therapist placed an open hand on either side of the participant’s face without touching to orient their face toward the therapist. Reinforcement was delivered in the form of positive verbal statements about the specific behavior he/she was teaching after each correct response (e.g., “You’re sitting so quietly, thank you for getting ready!... Thanks for looking at my eyes, I can tell you’re trying hard…You waited for me to start, nice work!”).

Prior to each treatment session, the participant was asked what they would like to earn from the leisure items available. Each time a correct response occurred (i.e., the duration of social gaze met criterion of the percentile schedule described below), a token was awarded on the token board (a magnetic erase board). A total of approximately 10 tokens had to be earned before the participant was awarded a 5-min break with their preferred item. Before beginning again, the board was erased, and the count restarted. As the participant earned tokens, he was reminded of his chosen reinforcer periodically and of how many more tokens would be needed to reach the goal of 10 tokens. If the participant ended a session before earning all 10 tokens, the tokens would carry over to the next session. One-hour sessions of 40 *look while listening* training trials were alternated with 1-h sessions of 40 *look while speaking* trials until the participant had received the requisite number of trials over the 2 days.

#### Behavior management

To ameliorate the potential for problem behaviors, the therapist employed a variety of behavioral techniques including planned ignoring, response blocking, and differential reinforcement procedures, on an as-needed basis for each participant. Trials were not started until problem behavior had ceased. If the participant began to show evidence of fatigue, the therapist used verbal encouragement, praising the participant for their hard work, and reminding them about how many more tokens were needed to earn their next break. In addition to blocking and redirecting any problem behavior, participants were praised and rewarded for engaging in appropriate behavior. For example, if the child was waiting with appropriate body posture, the therapist would note “I love how nicely you are sitting and waiting right now. Thanks for being so patient.”

#### Response measurement

During baseline and training trials of DTI, an observer recorded social gaze duration using the Percentile Schedule Software Program described in Hall et al. [32]. In order to determine precisely when the participant was coordinating eye gaze with the examiner, the observer sat in a chair directly to the right of the participant approximately 3 ft away and remained as unobtrusive as possible. Social gaze was defined as the child orienting his head toward the therapist so that his eyes looked directly at the therapist’s face. The observer pressed the “onset” button on the program when the child began to look at the therapist and pressed the “offset” button on the program when the child stopped looking at the therapist. During training trials, the computer program operated a percentile reinforcement schedule to determine whether the response should be reinforced [37].

The percentile schedule required two parameters to be pre-specified: the probability of reinforcing a criterion response (*w*) and the number of prior observations (*m*) to be included in the calculation. The percentile schedule equation, *k* = (*m* + 1) (1 - *w*), specifies the *k*th rank that must be exceeded before the current response became a criterion response. In this study, we set *w* = .5 and *m* = 10; thus, *k* = 5.5. The value of the current response would therefore be considered a criterion response only if it exceeded 5 of the previous 10 response values (i.e., the median response value). The criterion response would then have a 50% chance of being reinforced. The starting criterion for the percentile schedule was the duration of social gaze obtained during the baseline trials.

On each trial, if the participant engaged in social gaze within 5 s, the observer recorded the duration of social gaze on the computer. Once social gaze had been recorded, the computer simultaneously determined whether the therapist should deliver reinforcement with a random probability of 0.5. If a reinforcer was scheduled, the computer displayed a message to the observer stating, “Please reinforce now!” as a cue for the therapist to deliver a token. The therapist then provided positive reinforcement such as “Good job keeping eye contact,” then rewarded a token on the board. If the participant did not engage in social gaze within 5 s of the stimulus, the observer recorded 0 s for that trial, and the therapist delivered the graduated prompting procedure described above until social gaze was obtained. To indicate to the therapist that prompting or reinforcement was scheduled, the observer operating the Percentile Schedule Software Program pointed to a card containing a green circle to signal that reinforcement should be delivered, a yellow circle to signal that the participant’s social gaze did not meet the current criterion level and that prompting was required, and a red circle to signal that social gaze did not occur and that the prompting procedure was required. Prompts were delivered prior to the onset of the next trial, and for the “red circle” prompting procedure, the therapist followed a least-to-most prompt hierarchy until social gaze was achieved which marked the onset of the next trial. In conjunction with recording social gaze using the Percentile Schedule Software Program, the observer recorded on a data sheet whether the criterion for social gaze was met on each trial, and if any prompts or tokens were administered on a trial.

#### Treatment fidelity

To evaluate the fidelity of treatment implementation, we utilized the Teacher Performance Rate and Accuracy Scale (TPRA) created by Ross, Singer-Dudek, and Greer [38]. The TPRA examines three-term contingencies presented by a therapist to assess functional interrelationships between therapist antecedent (e.g., a verbal cue), student behavior (e.g., social gaze), and therapist consequence (e.g., verbal praise). We chose to utilize the TPRA because it offers an efficient method to directly measure and evaluate a therapist’s accuracy of instruction. Fidelity of treatment implementation was evaluated using the TPRA scale for a minimum of 40 trials per therapist across three therapists. Each of the three therapists demonstrated a score of 100% accuracy. Social gaze duration was automatically recorded by the eye tracker during the social challenge.

#### Inter-observer agreement

During DTI training, inter-observer agreement on the duration of social gaze was checked for 12.5% of the trials for each participant. On inter-observer agreement trials, the therapist simultaneously recorded the duration of the child’s social gaze using a stopwatch and noted the duration on a datasheet. Agreement between the observer and the therapist was computed using the intra-class correlation (ICC) coefficient computed across trials for each participant. The mean ICC across participants was 0.98 (range = 0.95 to 1.00) indicating that the agreement between observers was excellent.

### Data analysis

To provide a measure of improvement in social gaze across treatment, we superimposed Theil-Sen slopes onto the data for each participant. The Theil-Sen slope (also known as Kendall’s slope or the Nonparametric linear regression slope), is an alternative to the standard linear regression slope and has been recommended for use with single-subject data given that any outliers can significantly impact the standard linear regression slope [39]. The slope values were calculated using the Theil-Sen calculator available at http://www.singlecaseresearch.org/calculators/theil-sen [40]. In this context, a positive slope would indicate that the child’s social gaze duration improved across trials whereas a negative slope would indicate that social gaze duration deteriorated across trials. The significance of each slope was determined by computing a *z* score [39] with the alpha level set at 0.05.

All eye-tracking data were analyzed using Tobii Studio (version 3.4.8). For each participant, social gaze was quantified for all fixations that fell within a 52 cm × 28 cm box encompassing the therapist’s face. A fixation was defined using the I-VT Fixation Filter in Tobii Studio. The filter interpolated over 100 ms gaps to account for temporary loss of data (e.g., blinking and reflections), and all fixations shorter than 60 ms were discarded. Heart rate data collected during eye tracking were filtered through the built-in Polar algorithm which smoothed over abnormalities such as double and missed beats. The data were then averaged across the 5-min period of social interaction. All other analyses were conducted using SPSS Version 20 (SPSS, Inc).

## Results

### Social gaze duration during treatment

**Fig. 1:**
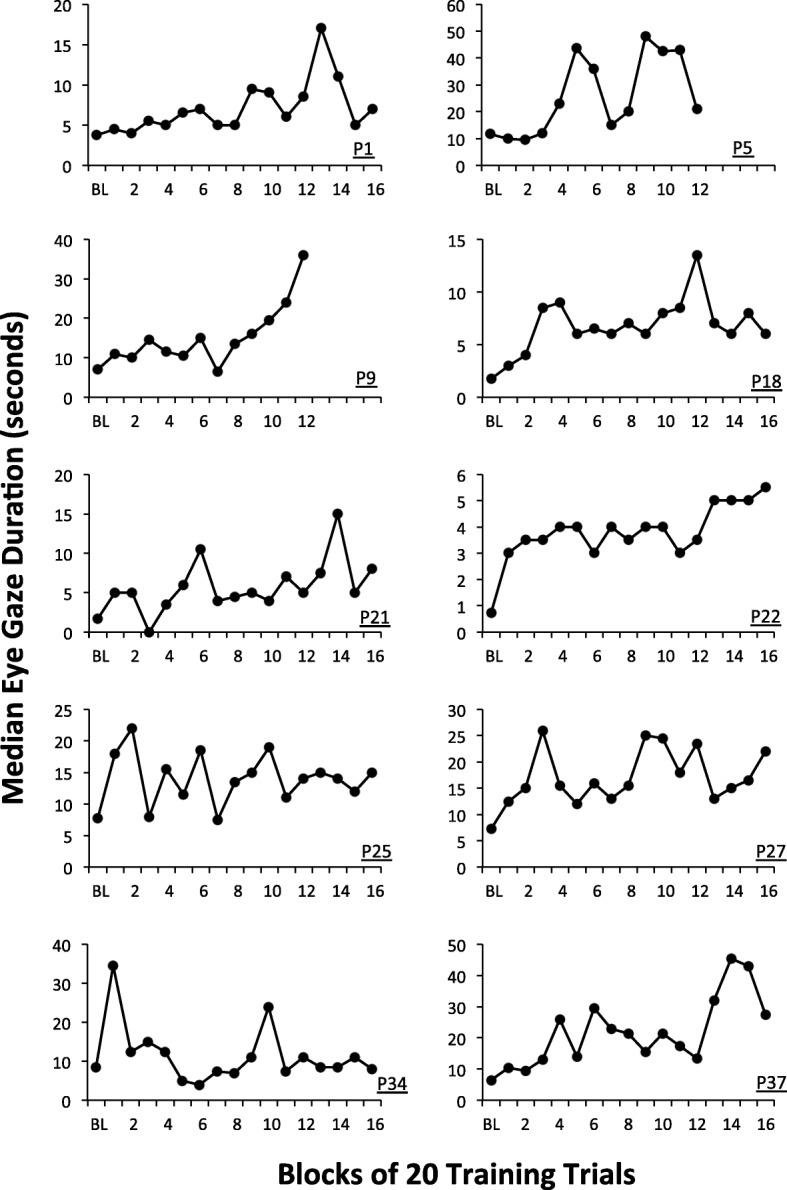
Median duration of social gaze observed across blocks of 20 trials for participants randomized to the *high-dose* group. Note the different *y*-axis scales

**Fig. 2:**
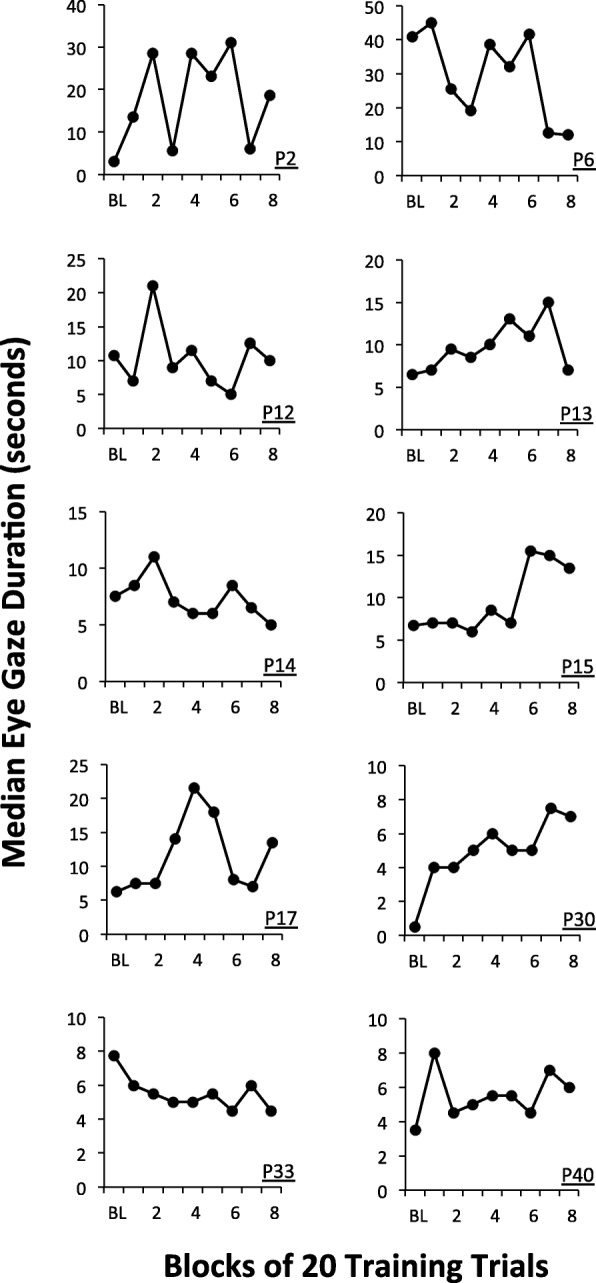
Median duration of social gaze observed across blocks of 20 trials for participants randomized the *low-dose* group. Note the different *y*-axis scales

Figure 1 shows the median duration of social gaze observed across blocks of 20 training trials for participants randomized to the high*-*dose group. Figure 2 shows the corresponding data for each participant randomized to the low*-*dose group.

There was a significant variability across participants in terms of the extent to which social gaze improved during training in each group (note the different *y*-axis scales). As described above, to quantify this variability, we superimposed a Thiel-Sen regression line onto the data for each participant and computed the slope of each regression line and its associated *p* value. The slope values for the Thiel-Sen regression lines and associated *p* values are shown in Table 2 for each participant in each group.

**Table 2:** Thiel-Sen regression slopes and standard errors (SE) computed for participants randomized to each group

		*Thiel-Sen regression*			
Group	Participant	Slope	SE	*z*	*p*
High dose	P1	0.33	0.12	2.84	0.005*
	P5	1.69	0.73	2.32	0.020*
	P9	1.28	0.42	3.05	0.002*
	P18	0.26	0.16	1.61	0.108
	P21	0.25	0.10	2.43	0.015*
	P22	0.14	0.05	3.05	0.002*
	P25	0.00	0.00	0.08	0.934
	P27	0.41	0.26	1.61	0.108
	P34	− 0.15	0.20	− .74	0.458
	P37	1.50	0.51	2.92	0.003*
Low dose	P2	0.77	0.82	0.94	0.348
	P6	− 2.43	1.45	− 1.67	0.095
	P12	− 0.23	0.75	− 0.31	0.755
	P13	0.82	0.41	1.98	0.048*
	P14	− 0.33	0.20	− 1.67	0.095
	P15	0.89	0.45	1.98	0.048*
	P17	0.46	0.49	0.94	0.348
	P30	0.60	0.22	2.71	0.007*
	P33	− 0.25	0.13	− 1.88	0.061
	P40	0.25	0.20	1.25	0.211

*SE* standard error

A positive slope indicates that social gaze duration improved across trials whereas a negative slope indicates that social gaze duration deteriorated across trials. The associated *z* score and significance of each learning slope is also given

*p < .05

The data in Table 2 shows that, for the boys who received high*-*dose treatment, six (60%) boys evidenced a significant improvement in social gaze duration across the training. Conversely, for boys who received low*-*dose treatment, three (30%) boys evidenced a significant improvement in social gaze duration across the training. The mean *z* score for participants in the high*-*dose group was 1.92 (SD = 1.31) whereas the mean *z* score for participants in the low*-*dose group was .43 (SD = 1.70). Comparison of the *z* scores indicated that boys who received high*-*dose treatment evidenced significantly greater improvements in social gaze compared to those who received low*-*dose treatment (*t*(18) = 2.2, *p* = .04, Cohen’s *d* = 0.98).

### Effect of ASD severity/diagnosis on treatment response

The mean comparison severity score on the ADOS-2 was 6.8 (SD = 2.2) for boys who responded to treatment and 6.4 (SD = 2.7) for non-responders, a non-significant difference between the groups (*t*(18) = .34, *p* = .74). Of the 15 participants who had an ASD diagnosis, 7 were treatment responders and 8 were non-responders, a non-significant difference between the groups (*p* = .60, Fisher’s exact test). There was therefore no effect of ASD severity or diagnosis on treatment response across treatment groups.

### Effect of treatment on social gaze and heart rate levels during the social challenge

**Fig. 3:**
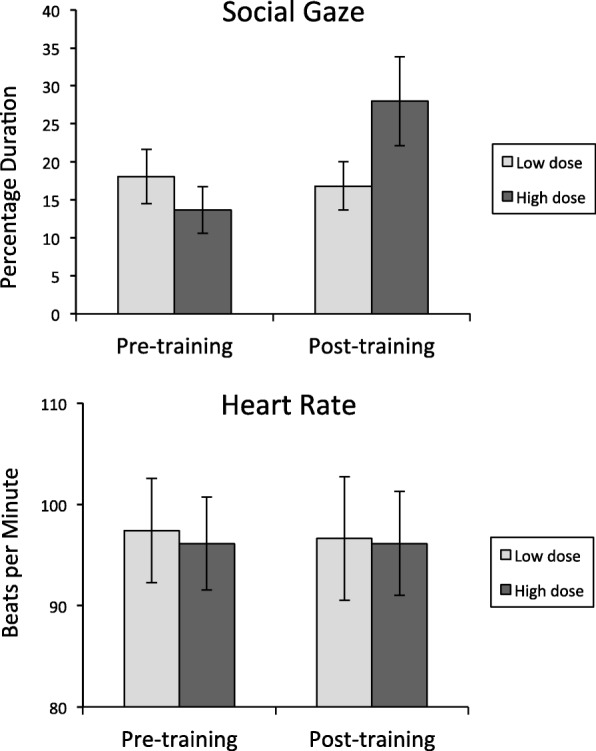
Upper panel: Mean percentage duration of social gaze recorded on the Tobii X120 eye tracker before and after treatment for participants randomized to each group. Lower panel: Mean heart rate recorded before and after treatment for participants randomized to each group. Error bars represent ± 1 standard error of the mean (SEM)

Figure 3 (upper panel) shows the mean percentage of social gaze recorded on the eye tracker before and after treatment for each group.

The mean percentage change in social gaze levels recorded from pre- to post-treatment was 14.4% (SD = 18.11%) for boys who received the high-dose treatment and − 1.3% (SD = 7.7%) for boys who received the low-dose treatment, a significant difference between the groups [*t*(17) = 2.40, *p* = .028, Cohen’s *d* = 1.13]. These data indicated that boys who received the high-dose treatment evidenced significantly greater improvements in social gaze compared to boys who received the low-dose treatment.

Figure 3 (lower panel) shows the corresponding heart rate data. The mean change in heart rate levels recorded from pre- to post-treatment was 0.0 bpm (SD = 11.53 bpm) for boys who received the high-dose treatment and − .79 bpm (SD = 7.62 bpm) for boys who received the low-dose treatment, a non-significant difference between the groups [*t*(15) = .17, *p* = .87, Cohen’s *d* = 0.08]. These data indicate that there were no differences between the groups in terms of the effect of treatment on heart rate levels.

## Discussion

Social gaze is arguably one of the most important skills required for appropriate social interaction. Previous research suggests that engaging in appropriate levels of social gaze during social interactions with others is a critical prerequisite for social engagement [4]. Accordingly, it is possible that exhibiting increased levels of social avoidance during social interactions may compound delays in social skills. Although social gaze avoidance is a pervasive behavior in boys with FXS, targeted interventions designed to ameliorate this behavior have not been developed. This may, in part, be due to the opinion held by some professionals in the field that social gaze training may not be beneficial for children with FXS and should be discouraged due to concern for potentially increased anxiety and/or problematic behavior in these children [17, 18, 41].

In this proof of concept study, building on previous research conducted by Hall and colleagues [32], we evaluated whether a targeted behavioral treatment could be employed to facilitate social gaze behavior in boys with FXS. To date, behavioral interventions designed to ameliorate these intransigent and socially stigmatizing behaviors in FXS have not been evaluated, particularly in the late childhood/early adolescence period when social gaze avoidance may become established in the child’s repertoire. This developmental period can thus present a significant challenge to boys with FXS.

Over the course of treatment, social gaze duration improved significantly for six of the ten boys who received high-dose treatment and for three of the ten boys who received low-dose treatment. Boys who received a greater number of DTI trials therefore benefited more from the treatment. To determine whether the skills learned during the treatment generalized to a naturalistic social setting, we also administered a social challenge in which the participant was required to engage in a conversation with an examiner. To provide an objective measure of social gaze behavior during the social challenge, an eye tracker positioned between the participant and the examiner simultaneously recorded social gaze levels of the participant. Results of this generalization test indicated that social gaze increased to a greater extent in those who had received the high*-*dose treatment compared to those who had received the low*-*dose treatment. Physiological arousal levels recorded simultaneously on a heart rate monitor did not appear to be impacted by the treatment.

There are several advantages to the design of the study presented here. First, the intervention utilized is an established and empirically supported behavioral training procedure designed to promote social gaze with a therapist. A further advantage of this study is the use of a percentile schedule, which provided consistent and reliable reinforcement, adapted to the child’s previous performance level. This resulted in a personalized delivery of reinforcement that took into account baseline levels of social gaze and enabled more precise shaping of the participant’s behavior.

There are also several limitations. First, the intervention required two researchers to be present in the room with the participant, a therapist to implement the treatment and another observer to operate the computerized percentile schedule and to record the duration of eye gaze. This may limit the ability for the procedure to be replicated in certain clinical settings. Second, neither the therapist nor the observer was blind to each participant’s group assignment, which may have affected measures of eye gaze duration recorded. Third, the current intervention may need to be adapted to better accommodate the needs of minimally verbal and non-verbal individuals. Furthermore, fatigue experienced by some of the participants over the course of the 2 days of treatment may have resulted in an eventual decreased rate of learning. Anecdotally, it appeared that boys who exhibited the highest levels of social gaze at baseline quickly became fatigued during the intervention and began to exhibit increased numbers of escape behaviors or simply “gave up” trying to look at the therapist. For these participants, it seems likely that more naturalistic forms of social gaze training, such as the methods employed by Carbone et al. [5] could be used rather than discrete trials.

An additional limitation is that there was no control to examine the effectiveness of relaxation training; rather, all participants regardless of treatment condition received similar amounts of relaxation training. Due to the absence of this control, future research comparing the impact of DTI with and without relaxation training may be helpful. Lastly, the short-term nature of the treatment, and the absence of a parent-training component, may limit the generalizability of the results.

Although DTI has a solid empirical foundation, we acknowledge that there is currently increased emphasis on the promise of naturalistic developmental behavioral interventions, which are still based on the principles of ABA, but are administered within a more naturalistic framework. It is possible that these approaches may have resulted in better generalization and less problem behavior (given that they are believed to be less stressful for the child). A lengthier follow-up study, perhaps over weeks or months, that utilizes alternative more naturalistic ABA intervention methods may therefore be warranted.

Further data could be collected to strengthen the findings of the current study such as using the eye tracker to record social gaze behavior automatically during the intervention [14]. Interestingly, Miller and colleagues [42] assessed whether a computer-assisted instruction (CAI) package (pairing visual and vocal stimuli) improved social gaze in three children with ASD. The CAI involved showing the participant a digital picture of a familiar adult accompanied by an audio recording of them saying “look at me!”, while the participant’s social gaze was tracked with an infrared camera. If the participant successfully made eye contact with the face on the computer screen within 5 s, the participant was provided a reinforcing item. Results demonstrated both significant increases in social gaze duration and decreased latency from adult vocalization to making eye contact across participants. These findings may support the future use of CAI to teach social gaze and interpersonal social skills in children with FXS. Future studies could also investigate the extent to which younger children with FXS are amenable to the intervention, before social gaze avoidance becomes established in the child’s repertoire. Studies could also examine whether integrating parent training into the intervention, rather than relying on trained therapists, results in greater generalizability of learned skills.

## Conclusions

The data reported in this proof of concept study show that improvements in social gaze behavior can be obtained in boys with FXS following short-term administration of a standardized behavioral intervention. The positive results should encourage other investigators to pursue further experimental studies based on the procedures reported in this paper. Although further research will be needed to determine whether these gains in social gaze can be generalized and maintained over time, overall, these data suggest that professionals should not be deterred from providing much needed targeted interventions for children with FXS.

## Abbreviations

ADOS: Autism Diagnostic Observation Schedule
ASD: Autism spectrum disorder
CAI: Computer-assisted instruction
DTI: Discrete trial instruction
ECAS: Eye Contact Avoidance Scale
FXS: Fragile X syndrome
IQ: Intelligence quotient
MSWO: Multiple stimulus without replacement
PoC: Proof of concept
TPRA: Teacher Performance Rate and Accuracy Scale
